# A randomised, placebo-controlled trial of anti–interleukin-1 receptor 1 monoclonal antibody MEDI8968 in chronic obstructive pulmonary disease

**DOI:** 10.1186/s12931-017-0633-7

**Published:** 2017-08-09

**Authors:** Peter M. A. Calverley, Sanjay Sethi, Michelle Dawson, Christine K. Ward, Donna K. Finch, Mark Penney, Paul Newbold, René van der Merwe

**Affiliations:** 1 School of Ageing and Chronic Disease, University of Liverpool, Liverpool, UK; 2 Division of Pulmonary, Critical Care and Sleep Medicine, University of Buffalo, State University of New York, Buffalo, NY USA; 3 MedImmune, Cambridge, UK; 4 MedImmmune, Gaithersburg, MD USA; 5 Present address: Bristol-Myers Squibb, Princeton, NJ USA; 6 Present address: UCB Pharma, Slough, UK; 7 Clinical Science Centre, University Hospital Aintree, Longmoor Lane, Liverpool, L9 7AL UK

**Keywords:** C-reactive protein, COPD, Fibrinogen, Interleukin-1 receptor 1, MEDI8968, Neutrophils, Pharmacology

## Abstract

**Background:**

Interleukin-1 receptor 1 (IL-1R1) inhibition is a potential strategy for treating patients with chronic obstructive pulmonary disease (COPD). MEDI8968, a fully human monoclonal antibody, binds selectively to IL-1R1, inhibiting activation by IL-1α and IL-1β. We studied the efficacy and safety/tolerability of MEDI8968 in adults with symptomatic**,** moderate-to-very severe COPD.

**Methods:**

This was a phase II, randomised, double-blind, placebo-controlled, multicentre, parallel-group study. Subjects aged 45–75 years and receiving standard maintenance therapy with ≥2 exacerbations in the past year were randomised 1:1 to receive placebo or MEDI8968 300 mg (600 mg intravenous loading dose) subcutaneously every 4 weeks, for 52 weeks. The primary endpoint was the moderate/severe acute exacerbations of COPD (AECOPD) rate (week 56 post-randomisation). Secondary endpoints were severe AECOPD rate and St George’s Respiratory Questionnaire-COPD (SGRQ-C) score (week 56 post-randomisation).

**Results:**

Of subjects randomised to placebo (*n* = 164) and MEDI8968 (*n* = 160), 79.3% and 75.0%, respectively, completed the study. There were neither statistically significant differences between treatment groups in moderate/severe AECOPD rate ([90% confidence interval]: 0.78 [0.63, 0.96], placebo; 0.71 [0.57, 0.90], MEDI8968), nor in severe AECOPD rate or SGRQ-C scores. *Post-hoc* analysis of subject subgroups (by baseline neutrophil count or tertiles of circulating neutrophil counts) did not alter the study outcome. The incidence of treatment-emergent adverse events (TEAEs) with placebo and MEDI8968 treatment was similar. The most common TEAE was worsening of COPD.

**Conclusions:**

In this phase II study, MEDI8968 did not produce statistically significant improvements in AECOPD rate, lung function or quality of life.

**Trial registration:**

ClinicalTrials.gov, NCT01448850, date of registration: 06 October 2011.

**Electronic supplementary material:**

The online version of this article (doi:10.1186/s12931-017-0633-7) contains supplementary material, which is available to authorized users.

## Background

Chronic obstructive pulmonary disease (COPD) is a progressive inflammatory disease of the lower airways, characterised by persistent airflow limitation [1]. Acute exacerbations of COPD (AECOPD) cause a persistent deterioration of the patients’ quality of life [2]. Once hospitalisation occurs, patients often require repeated hospital admissions, with deterioration of COPD leading to eventual death [3].

Interleukin (IL)-1α and IL-1β are pro-inflammatory cytokines produced by a variety of immune and stromal cells [4] and are thought to play an important role in the pathophysiology of COPD. In patients with COPD, IL-1β is elevated in sputum, serum and bronchoalveolar lavage fluid [4] and, along with IL-1α, is elevated both in patients with stable disease and those experiencing exacerbations [5]. Furthermore, both cytokines are released in response to cigarette smoke [4, 6], the most common risk factor for COPD [1]. IL-1α and IL-1β bind to the IL-1 receptor 1 (IL-1R1) [4, 7]. This leads to stimulation of neutrophilic inflammation, which is associated with the narrowing of small airways [6, 8] and the release of other cytokines that sustain the inflammatory response. IL-1β concentrations in sputum from patients with COPD correlate with concentrations of sputum neutrophils and sputum IL-8, and are inversely correlated with forced expiratory volume in 1 s (FEV_1_) [9, 10]. Sputum IL-1β concentrations are further increased in patients with AECOPD and show a strong correlation with bacterial infections [11]. Animal models of smoke exposure have shown that inflammation of the airways is dependent on IL-1R1 signalling [7]. In particular, IL-1α is a crucial mediator of neutrophilic inflammation [6], with inhibition of IL-1β reducing this inflammation [12]. Additionally, chronic inflammation may increase susceptibility to bacterial and viral infections that are the major cause of AECOPD [13, 14]. Inhibition of IL-1R1 using anakinra resulted in a reduction of airway neutrophilia in a human lipopolysaccharide challenge model of lung inflammation in a small number of healthy volunteers [15]. Inhibition of IL-1R1 represents a potential strategy for the treatment of patients with COPD by blocking the effects of both IL-1α and IL-1β.

MEDI8968, a fully human immunoglobulin G2 monoclonal antibody that binds selectively to IL-1R1 to inhibit its activation by IL-1α and IL-1β, has been investigated in subjects with rheumatoid arthritis [16] and osteoarthritis [17]. We hypothesised that inhibition of IL-1R1 would reduce neutrophilic airway inflammation in subjects with stable COPD, resulting in a reduced frequency and severity of AECOPD. To test this hypothesis, we undertook a phase II study to examine the efficacy and safety/tolerability of MEDI8968 in adults with moderate-to-very severe COPD, with the primary objective of examining whether the frequency of moderate/severe AECOPD was reduced. In addition, we evaluated the response to MEDI8968 in subject subgroups defined by C-reactive protein (CRP), fibrinogen and, in *post-hoc* analyses, blood neutrophil counts.

## Methods

### Subjects

We enrolled subjects aged 45–75 years with symptomatic, moderate-to-very severe COPD (Global Initiative for Chronic Obstructive Lung Disease [GOLD] stage II–IV [1]), receiving standard maintenance therapy and who had ≥2 AECOPD that required oral corticosteroids, antibiotics or hospitalisation in the 12 months prior to screening. Full inclusion, exclusion and study-stopping criteria are listed in the online Additional file 1.

### Study design

This was a phase II, randomised, double-blind, placebo-controlled, multicentre, parallel-group study (CP1103; ClinicalTrials.gov NCT01448850), conducted at 68 sites in Bulgaria, Czech Republic, Hungary, Latvia, Lithuania, Philippines, Poland, Ukraine, United Kingdom and United States. The study consisted of a 17–23-day run-in period (visits 1–3) and a 52-week treatment period (visits 4–19; weeks 1, 4, 5, 8, 9 and every 4 weeks [Q4W] thereafter until week 53). Subjects returned to the clinic 8 weeks (week 61) and 16 weeks (week 69) after the treatment period, for follow-up visits (visits 20–21).

During screening, FEV_1_ measurements determined the standard maintenance care therapy (budesonide/formoterol or tiotropium or budesonide/formoterol plus tiotropium), which replaced the existing maintenance therapy and was assigned for each subject at the start of run-in (online Additional file 1). Following screening/run-in, subjects were randomised 1:1 to receive placebo or MEDI8968 as a 600 mg intravenous (IV) dose on day 1 (loading dose), followed by 300 mg subcutaneous (SC) (two 150 mg injections) Q4W, for a total of 14 doses. The single 600 mg IV infusion was administered over a minimum of 1 h (for further details on randomisation and blinding, see the online Additional file 1).

### Assessments

The primary endpoint was the annualised rate of moderate/severe AECOPD, including data up to week 56, summarised as a per-person-per-year rate (measured at all visits during treatment and follow-up). An AECOPD was defined as worsening of ≥2 major symptoms (dyspnoea, sputum volume, sputum purulence) or worsening of one major and one minor symptom (sore throat, cold, fever without other cause, increased cough or wheeze) for ≥2 consecutive days [18]. The severity of AECOPD was categorised based on the treatment required: increase in normal therapy, antibiotics/systemic corticosteroids or hospitalisation for mild, moderate or severe AECOPD, respectively. Additionally, the moderate/severe AECOPD rate was compared between subjects by baseline CRP (≥0.347 mg/dL cut-off; inclusion criterion for a study of canakinumab in COPD [19]) and fibrinogen (≥ median cut-off) concentrations as part of a pre-specified analysis.

Secondary endpoints included severe AECOPD rate and change from baseline in SGRQ-C total and symptom domain scores (measured at weeks 1, 5, 13, 25, 37, 53 and 69) [20, 21].

Exploratory endpoints included change from baseline in pre-bronchodilator FEV_1_ and change from baseline in Exacerbations of Chronic Pulmonary Disease Tool Respiratory Symptoms (E-RS) total score (online Additional file 1) and exploratory serum biomarker analyses were performed using Rules Based Medicine and in house assays. Pharmacokinetic (PK) and immunogenic profile measurements are included in the online Additional file 1. Safety and tolerability were assessed throughout the treatment and follow-up periods (online Additional file 1).

A *post-hoc* analysis was carried out to evaluate the rate of moderate/severe AECOPD, as described for the primary endpoint, and change from baseline in SGRQ-C scores by baseline blood neutrophil counts and tertiles. The tertiles comprised subjects with baseline neutrophil counts of ≤4.27 × 10^3^ cells/μL (1st tertile), >4.27 × 10^3^–≤5.68 × 10^3^ cells/μL (2nd tertile) and >5.68 × 10^3^ cells/μL (3rd tertile). Time to first severe-only AECOPD was also analysed post hoc.

### Statistics

Efficacy analyses were conducted using the modified intention-to-treat (mITT) population, which comprised all randomised subjects who received any study drug. The safety population included all randomised subjects who received at least one dose of the study drug. An interim analysis was carried out at 26 weeks. A total of 134 subjects per arm was required to detect a 40% reduction in the rate of moderate/severe AECOPD at an interim analysis performed after 26 weeks of treatment, assuming 80% power and alpha of 10%. Accounting for a dropout rate of 15%, and subjects contributing partial data to the primary endpoint, the number of randomised subjects was increased to 150 per arm. The AECOPD rate was analysed using a Poisson regression model adjusted for over dispersion, with number of exacerbations as the outcome and the log of follow-up time as an offset variable, with covariates for treatment group, background maintenance therapy and previous exacerbations. The adjusted mean rates, treatment ratio, 90% confidence interval (CI) and *p*-value for the comparison of MEDI8968 versus placebo have been presented. The change from baseline in continuous endpoints was analysed using mixed model repeated measures, including treatment, visit and treatment-by-visit interaction, and baseline score and background maintenance therapy as covariates.

## Results

### Subjects

**Fig. 1:**
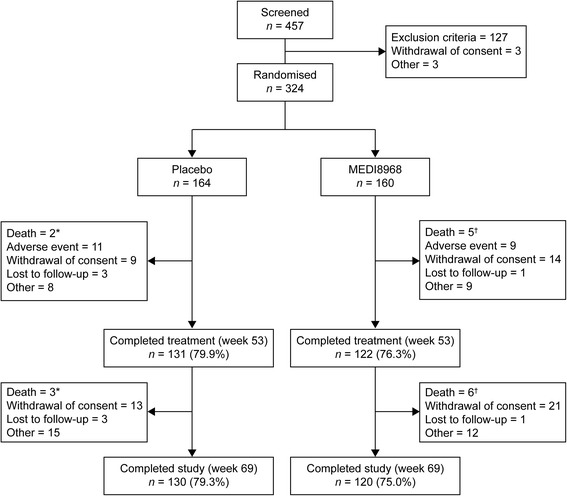
Subject disposition *Due to metastatic lung cancer, cardiopulmonary failure and cardiac failure. ^†^Due to pneumonia, metastatic neoplasm, necrotising pancreatitis, cerebral haemorrhage, dyspnoea and pulmonary embolism

Between 11 November 2011 and 18 February 2014, 324 subjects were randomised (*n* = 164 placebo; *n* = 160 MEDI8968) and formed the mITT and safety populations. The study ended as planned after subjects completed the final visit. Overall, 250 subjects (77.2%) completed the study (79.3% placebo; 75.0% MEDI8968) (Fig. 1). Demographics and baseline disease characteristics were similar between treatment groups (Table 1).

**Table 1:** Demographics and baseline disease characteristics (ITT population)^a^

		Placebo (*n* = 164)	MEDI8968 (*n* = 160)	Total (*N* = 324)
Age, years, mean (SD)		63.0 (6.8)	62.8 (6.7)	62.9 (6.8)
Male, *n* (%)		110 (67.1)	110 (68.8)	220 (67.9)
BMI, kg/m^2^, mean (SD)		25.8 (5.1)	25.7 (4.9)	25.7 (5.0)
Baseline GOLD 2001 status, *n* (%)	II	44 (26.8)	44 (27.5)	88 (27.2)
	III	90 (54.9)	79 (49.4)	169 (52.2)
	IV	30 (18.3)	37 (23.1)	67 (20.7)
FEV_1_, L, mean (SD)	Pre-bronchodilator	1.1 (0.4)	1.2 (0.5)	1.16 (0.5)
	Post-bronchodilator	1.2 (0.4)	1.3 (0.5)	1.24 (0.5)
FEV_1_ predicted, %, mean (SD)	Pre-bronchodilator	38.6 (12.7)	39.7 (14.1)	39.1 (13.4)
	Post-bronchodilator	42.1 (13.3)	41.9 (14.4)	42.0 (13.8)
SGRQ-C, mean (SD)	Total	58.9 (18.0)	61.2 (17.6)	60.0 (17.8)
	Symptom	72.4 (18.8)	74.4 (18.0)	73.4 (18.4)
CRP, mg/dL, *n* (%)	<0.347	84 (51.2)	84 (52.5)	168 (51.9)
	≥0.347	80 (48.8)	76 (47.5)	156 (48.1)
Fibrinogen, *n* (%)	< median	82 (51.6)	73 (46.2)	155 (48.9)
	≥ median	77 (48.4)	85 (53.8)	162 (51.1)

*BMI* body mass index, *CRP* C-reactive protein, *FEV*_*1*_ forced expiratory volume in 1 s, *GOLD* Global Initiative for Chronic Obstructive Lung Disease, *ITT* intention-to-treat, *SD* standard deviation *SGRQ-C* St George’s Respiratory Questionnaire-chronic obstructive pulmonary disease

^a^ITT population: all randomised subjects

### Efficacy

#### Exacerbations

Sixty-eight (41.5%) subjects in the placebo group and 65 (40.6%) in the MEDI8968 group experienced at least one moderate/severe AECOPD to week 56. Although there was a numerical reduction in the exacerbation rate with MEDI8968, the primary endpoint was not met, with placebo and MEDI8968 groups showing moderate/severe AECOPD rates (90% CI) to week 56 of 0.78 (0.63, 0.96) and 0.71 (0.57, 0.90), respectively. The treatment ratio (90% CI) for MEDI8968 versus placebo was 0.92 (0.68, 1.25), a relative annual exacerbation rate reduction (AERR) (90% CI) of 8% (−25%, 32%; *p* = 0.645) in the MEDI8968 group compared with placebo. The AECOPD rate in the placebo group was lower than expected at 0.78. However, the total number of patient-years included in the analysis had sufficient statistical power to detect a 40% reduction in moderate/severe AECOPD rate (determined by *post-hoc* power calculation based on placebo rate at the end of the study) and ruled out a reduction in exacerbation rate of more than 32%.

The severe AECOPD rate (90% CI) was 0.14 (0.09, 0.21) with placebo treatment and 0.10 (0.06, 0.16) with MEDI8968 treatment (treatment ratio [90% CI]: 0.71 [0.40, 1.28]; relative AERR [90% CI]: 29% [−28%, 60%]; *p* = 0.340). Twenty (12.2%) subjects in the placebo group and 16 (10.0%) in the MEDI8968 group had at least one severe AECOPD to week 56.

**Fig. 2:**
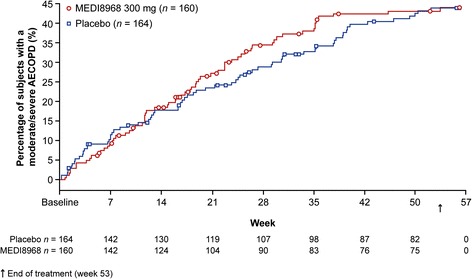
Kaplan-Meier plot of time to first moderate or severe AECOPD (mITT population). Kaplan-Meier method used to estimate the percentage of subjects with a moderate/severe AECOPD *AECOPD* acute exacerbations of chronic obstructive pulmonary disease, *mITT* modified intention-to-treat

**Fig. 3:**
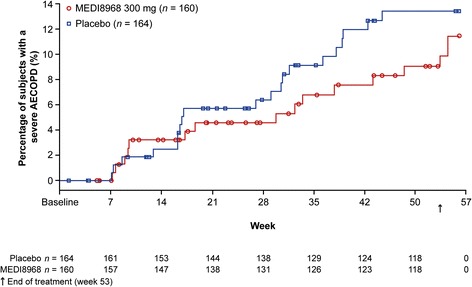
Kaplan-Meier plot of time to first severe AECOPD (mITT population). Kaplan-Meier method used to estimate the percentage of subjects with a severe AECOPD *AECOPD* acute exacerbations of chronic obstructive pulmonary disease, *mITT* modified intention-to-treat

There were no significant differences in time to first moderate/severe or severe-only AECOPD between treatment groups (moderate/severe: hazard ratio [90% CI]: 1.04 [0.78, 1.38], *p* = 0.824; severe: hazard ratio [90% CI]: 0.82 [0.47, 1.43], *p* = 0.562) (Figs. 2 and 3).

**Fig. 4:**
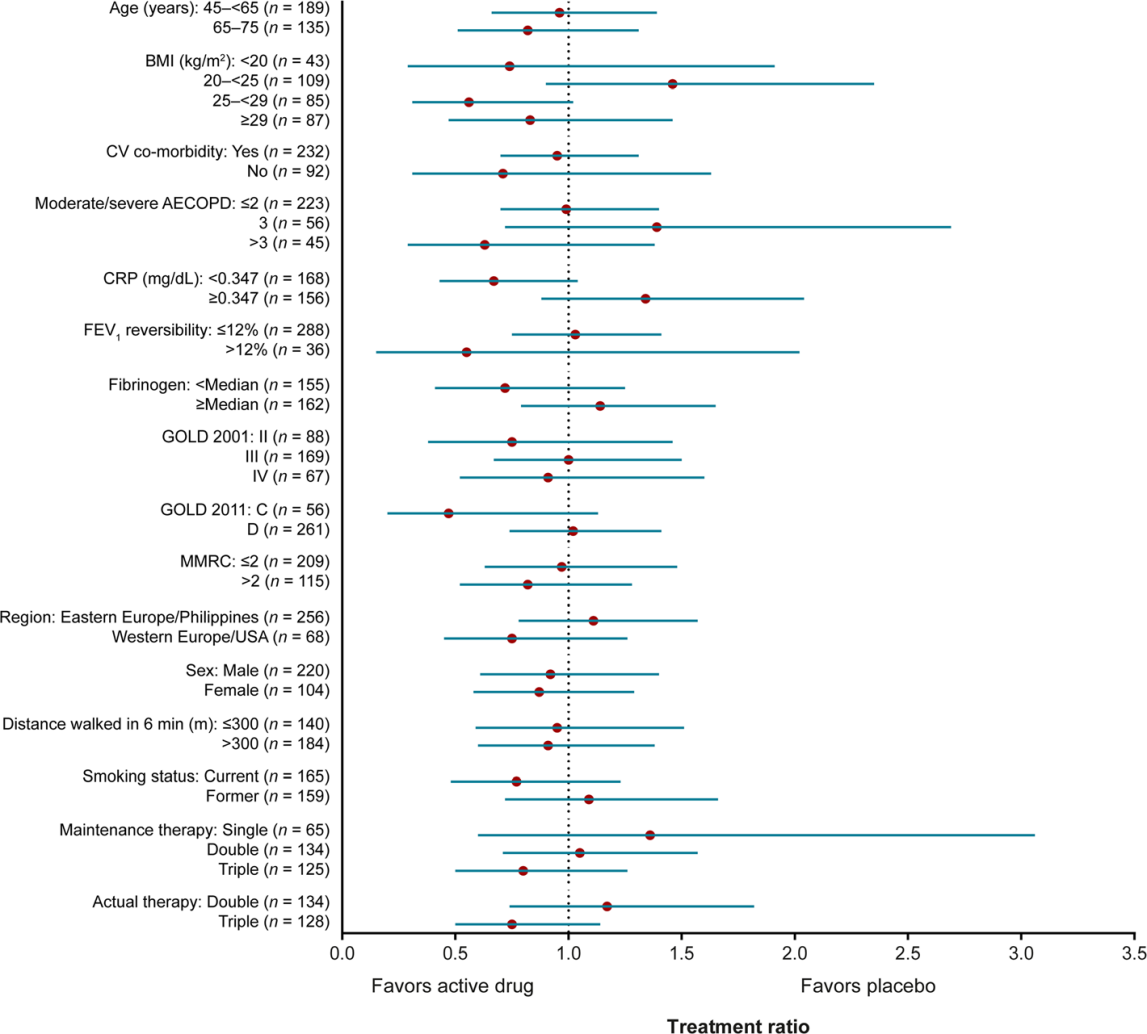
Forest plot of treatment ratio (90% CI) by subgroup (mITT population). Values <1 represent a reduction of AECOPD rate relative to placebo. Analysed using Poisson regression with Pearson correction, adjusting for treatment, background therapy and history of previous exacerbations *AECOPD* acute exacerbations of chronic obstructive pulmonary disease, *BMI* body mass index, *CI* confidence interval, *CRP* C-reactive protein, *CV* cardiovascular, *FEV*_*1*_ forced expiratory volume in 1 s, *GOLD* Global Initiative for Chronic Obstructive Lung Disease, *mITT* modified intention-to-treat, *MMRC* Modified Medical Research Council dyspnoea scale

Treatment with MEDI8968 did not have a significant effect on AECOPD rate in any of the pre-specified subgroup analyses (Fig. 4). Analyses of the moderate/severe AECOPD rate by baseline CRP (<0.347 mg/dL and ≥0.347 mg/dL) and fibrinogen (< median and ≥ median) concentrations showed no statistically significant differences between placebo and MEDI8968 groups (Additional file 1: Table S1 and Fig. 4).

#### Health-related quality of life and lung function

No significant differences were observed between treatment groups in St George’s Respiratory Questionnaire for COPD (SGRQ-C) change from baseline at week 53, for total score (mean difference [90% CI]: 0.54 [−1.99, 3.08]; *p* = 0.724) or symptom domain score (mean difference [90% CI]: –1.21 [−4.25, 1.83]; *p* = 0.511) (Additional file 1: Figure S1A and B). There were also no significant differences seen in change from baseline in FEV_1_ (Additional file 1: Table S2) and E-RS total score (Additional file 1: Figure S2).

#### Effect on blood neutrophil counts and serum CRP and fibrinogen

**Fig. 5:**
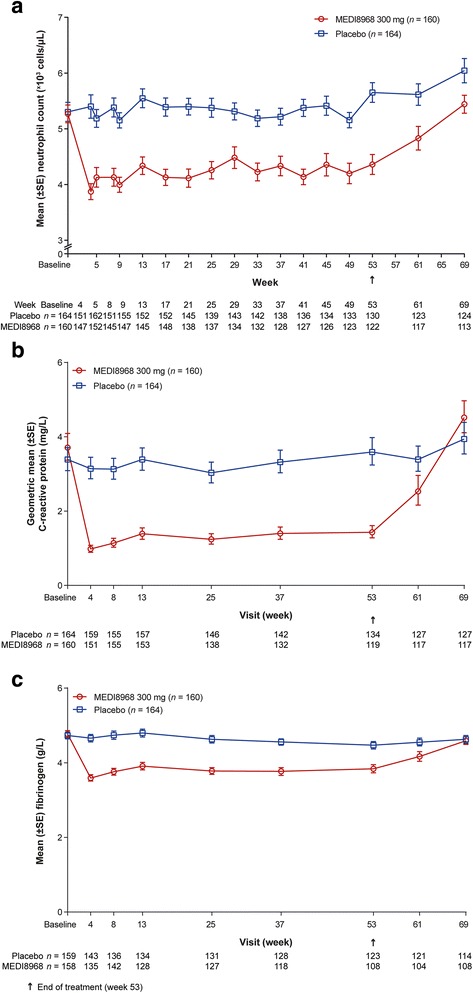
**a** Circulating neutrophil count, (**b**) serum CRP and (**c**) serum fibrinogen over time (safety population). Raw mean and standard errors estimated at each visit (neutrophils, panel **a**; fibrinogen, panel **c**). Raw geometric mean and standard errors estimated at each visit (CRP, panel **b**); the geometric mean was used as the distribution of the CRP data was skewed, thus requiring log transformation prior to calculation of the mean *CRP* C-reactive protein, *SE* standard error

No subjects had blood neutrophil counts below 1.5 × 10^3^ cells/μL, which is indicative of neutropenia [22], at baseline. Mean (standard deviation [SD]) blood neutrophil count at baseline was similar between treatment groups: 5.30 × 10^3^ (2.17 × 10^3^) cells/μL with placebo (*n* = 164) and 5.27 × 10^3^ (2.01 × 10^3^) cells/μL with MEDI8968 (*n* = 160). After the first dose, mean (SD) blood neutrophil count remained stable with placebo (5.40 × 10^3^ [2.58 × 10^3^] cells/μL; *n* = 151) and decreased in the MEDI8968 group (3.87 × 10^3^ [1.73 × 10^3^] cells/μL; *n* = 147). Throughout the treatment period, blood neutrophil counts in the MEDI8968 group remained consistently and significantly lower than the placebo group (*p* < 0.0012). Sixteen weeks after the last dose, mean (SD) neutrophil counts were 6.04 × 10^3^ (2.42 × 10^3^) cells/μL in the placebo group (*n* = 124) and had increased to 5.44 × 10^3^ (1.71 × 10^3^) cells/μL (*n* = 113) in subjects receiving MEDI8968 (Fig. 5).

There were statistically significant reductions from baseline throughout the treatment period in serum concentrations of CRP (*p* < 0.0001) and fibrinogen (*p* < 0.0001) in the MED8968 treatment group compared with placebo (Fig. 5).

#### Exacerbations and health-related quality of life by baseline peripheral blood neutrophil count

**Fig. 6:**
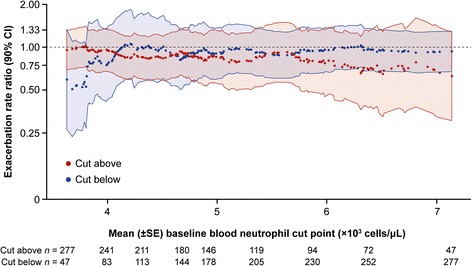
Treatment effect on rate of AECOPD by baseline neutrophil count (mITT population). Exacerbation rate ratio and 90% confidence interval estimated by bisecting the data at each value of the baseline blood neutrophils and analysing the data above and below the cut point using a negative binomial regression adjusting for treatment, background therapy and history of exacerbations *AECOPD* acute exacerbations of chronic obstructive pulmonary disease, *CI* confidence interval, *mITT* modified intention-to-treat, *SE* standard error

A *post-hoc* subgroup analysis of exacerbation rates was performed using baseline neutrophil counts. At high baseline neutrophil counts, there was a trend towards benefit in the treatment arm compared with placebo (Fig. 6). Additionally, a tertile analysis of baseline neutrophil counts assessing exacerbations (Additional file 1: Table S1) or SGRQ-C (Additional file 1: Figure S3) was consistent with these results; however, the treatment effect in the top tertile for neutrophil count was not statistically significant.

### Safety and tolerability

The incidence of treatment-emergent adverse events (TEAEs) was similar between treatment groups (*n* = 130 [79.3%] placebo; *n* = 130 [81.3%] MEDI8968), with the proportion of subjects with TEAEs considered treatment-related being low across both treatment groups (*n* = 8 [4.9%] placebo; *n* = 17 [10.6%] MEDI8968) (Additional file 1: Table S3). Overall, the most common TEAE was worsening of COPD (*n* = 76 [46.3%] placebo; *n* = 70 [43.8%] MEDI8968). Pneumonia occurred in eight subjects receiving placebo (4.9%) and ten receiving MEDI8968 (6.3%). The incidence of treatment-emergent serious adverse events (TESAEs) was similar with placebo (*n* = 35 [21.3%]) and MEDI8968 (*n* = 41 [25.6%]). There were nine deaths during the study (Fig. 1): three in the placebo group (due to metastatic lung cancer, cardiopulmonary failure and cardiac failure) and six in the MEDI8968 group (due to pneumonia, metastatic neoplasm, necrotising pancreatitis, cerebral haemorrhage, dyspnoea and pulmonary embolism); none were considered related to treatment.

There were no clinically important differences between the placebo and MEDI8968 groups in laboratory measures, vital signs and electrocardiogram parameters.

### Pharmacokinetic and immunogenicity profile

The PK population comprised 165 subjects (*n* = 7 placebo; *n* = 158 MEDI8968). After the 600 mg IV loading dose, the mean trough MEDI8968 serum concentration at week 4 in the active treatment participants was 63.4 ng/μL; after subsequent dosing with 300 mg SC Q4W, this value had approximately halved by week 53 to 28.6 ng/μL. Sixteen weeks after the last administered dose (week 69), the mean MEDI8968 serum concentration had decreased to 0.1 ng/μL, with most samples being below the limit of quantitation. Of the MEDI8968-dosed subjects, 19 (12.3%) were confirmed positive for anti-drug antibody (ADA), of whom nine (5.6%) were positive for neutralising antibodies (for further information on the immunogenicity profile, see the online Additional file 1).

## Discussion

This is the first study to investigate the efficacy and safety/tolerability of MEDI8968, an anti–IL-1R1 monoclonal antibody, in subjects with COPD. Despite being well tolerated, MEDI8968 (600 mg IV loading dose; 300 mg SC Q4W) did not produce statistically significant improvements in the rate of moderate/severe or severe-only AECOPD, time to first AECOPD, SGRQ-C total or symptom domain score or FEV_1_ in the total study population.

In this study population, MEDI8968 treatment was associated with a similar incidence of TEAEs and TESAEs as the placebo treatment. MEDI8968 decreased the neutrophil concentration after the first dose, an effect that persisted throughout the treatment period, but which was reversible following washout. This is consistent with the mechanism of action of MEDI8968 and the effect previously observed with MEDI8968 in osteoarthritis [17]. An increase in bacterial infections is a concern associated with the use of biologic therapies, due to potential immunosuppression [23, 24]. However, in our study, the incidence of pneumonia in MEDI8968-treated subjects was similar to that in the placebo group (*n* = 10 [6.3%] vs. *n* = 8 [4.9%]); furthermore, the overall highest incidence of infections was reported with placebo. It is possible this was, in part, due to the exclusion of subjects with neutrophil counts <2.5 × 10^3^ cells/μL at baseline.

MEDI8968 has previously been evaluated in subjects with rheumatoid arthritis [16] and osteoarthritis of the knee [17]; however, only modest improvements were observed in these studies. Specifically, MEDI8968 demonstrated statistically significant higher scores in ACR20/50 (20/50% improvement in American College of Rheumatology criteria) and Disease Activity Score 28–CRP protein compared with placebo in subjects with rheumatoid arthritis, but responses were weak or absent in swollen joints count and physician and patient global assessments [16]. In subjects with osteoarthritis of the knee, MEDI8968 did not demonstrate statistically significant improvements in pain [17].

PK analysis showed that MEDI8968 exposure concentrations were as expected for the dosing regimen used; the mean MEDI8968 trough concentration at week 4 (after loading dose) was approximately double that at week 53 (28.6 ng/μL), which was similar to the trough concentration previously observed with MEDI8968 300 mg SC Q4W in subjects with osteoarthritis of the knee (31.1 ng/μL) [17]. Moreover, MEDI8968 exposure was higher than in subjects with rheumatoid arthritis receiving 250 mg SC Q4W, in whom a statistically significant improvement was seen [16], and more than 10 times higher than the MEDI8968 concentration of 2.03 ng/μL that resulted in a 90% inhibition of IL-1R1 signalling in vitro. Suppression of neutrophil counts in the MEDI8968-treated group compared with placebo was consistent with achievement of pharmacologically active exposure concentrations.

In addition, it was considered that MEDI8968 exposure was affected by the presence of ADA in only nine (5.6%) MEDI8968-treated subjects. Consequently, inadequate exposure was not considered to be a significant factor affecting the outcome of the study. Nevertheless, a lack of sufficient pulmonary exposure cannot be ruled out since concentrations of MEDI8968 reaching the lung were not measured directly. If this were the case, it would be difficult to rectify, as subject exposure in this study was as high as practically possible.

For the primary analysis, the study was designed with sufficient power to detect a 40% reduction in the rate of moderate/severe AECOPD at a 26 week-interim analysis. The annual rate of moderate/severe AECOPD observed in the placebo group was lower than expected (0.78), and lower than that assumed in the power calculation of this study (1.27). Despite this, the study did have sufficient power to detect a 40% difference after all subjects had completed 52 weeks of treatment and ruled out a reduction in exacerbation rate of more than 32%. A smaller effect of MEDI8968 on AECOPD cannot be excluded and could be investigated further in another larger study, powered to detect a smaller effect size. A number of current therapies for COPD that are indicated to reduce exacerbations have small effect sizes, demonstrating a 14% reduction in exacerbations [25, 26].

Pre-specified subgroup analyses were based on common covariates, such as age, gender, severity of disease and smoking status, as well as subgroups relevant to the mechanism of action of MEDI8968, such as baseline CRP and fibrinogen concentrations. Serum CRP and fibrinogen concentrations have both been used as surrogate markers of inflammation in COPD, and plasma fibrinogen concentration is now an approved biomarker in COPD, although not at the time this study was conducted [27, 28]. However, none of these analyses identified a subgroup in which treatment with MEDI8968 had a significant effect on AECOPD rate, despite a clear, statistically significant impact of treatment on serum CRP and fibrinogen concentrations. As pharmacodynamic markers may also serve as predictive biomarkers of response [29], and IL-1 pathway activation has been clearly linked to neutrophilia pre-clinically in multiple animal models, we sought to evaluate efficacy of MEDI8968 in the subjects with COPD in this study by their baseline blood neutrophil counts. Neutrophils are thought to be a marker of future exacerbation risk [30]. This analysis suggests potential for a greater treatment effect with MEDI8968 at higher neutrophil baseline counts, but the numbers of subjects with higher baseline neutrophils were low and therefore no significant effect could be demonstrated when the analysis was performed using a tertile approach. It has been demonstrated that the simultaneous elevation of all three markers (neutrophils, CRP and fibrinogen) increases the risk of COPD exacerbations [31]. It could be expected that the inverse, the simultaneous reduction in all three markers, would reduce exacerbations. While we did not specifically evaluate this, it is likely that the majority of subjects who received MEDI8968 within this study experienced this combined reduction in neutrophil count and serum CRP and fibrinogen concentration, but with no subsequent impact on AECOPD rate.

The population of this study included subjects with symptomatic moderate-to-very severe COPD receiving standard maintenance therapy; it was hypothesised that targeting a more specific patient population may result in greater clinical benefits. COPD exacerbations are heterogeneous in their aetiology and different phenotypes respond differently to therapy [11]. Bacterial infections, together with viral infections, are responsible for the majority of exacerbations of COPD [5, 32]. The lower respiratory tract of 25–50% of patients with stable COPD is colonised by bacteria [14], which has been linked to disease progression [13].

An elevated concentration of sputum IL-1β is a marker for bacterial colonisation of the lower respiratory tract during stable disease [33] and for bacteria-associated exacerbations [11]. Furthermore, an elevated CRP concentration, a marker of systemic inflammation, can act as a potential biomarker for identifying patients with bacteria-associated AECOPD [34, 35]. This suggests that sputum IL-1β or serum CRP concentrations could be used as biomarkers for identifying patients with COPD whose exacerbations are associated with bacterial infection and who may benefit from anti–IL-1 treatment approaches. However, our data suggest that baseline CRP concentration is not sufficient to identify these individuals. Recognizing that a biomarker approach would be desirable for this type of treatment, we did attempt to identify novel serum biomarkers in this study. However, we did not reliably detect IL-1 in serum, nor identify any serum biomarkers that could be clearly linked to clinical response or even PD effects. This perhaps reflects a lack of down-stream biomarkers that are exclusively dependent on IL-1 within those that were measurable in the serum by the methods used, and possibly lack of sufficient power in the study to detect a biomarker-driven subgroup.

Several studies have suggested that IL-1α and IL-1β play an important role in COPD by initiating an inflammatory response, and that blocking their signalling through IL-R1 could lead to clinical benefits [4, 6, 7, 15]. However, when tested in COPD studies, anti-cytokine treatments (tumour necrosis factor-α inhibitors, anti–IL-8 therapies, the anti–IL-1β antibody canakinumab) have had little clinical efficacy, despite showing potential in pre-clinical models [4, 36–42]. This inability of anti-cytokine therapies, including MEDI8968, to produce clinical benefits in patients with COPD could be due to the functional redundancy in inflammatory signalling pathways involved in the disease [43].

A limitation of anti-cytokine studies in COPD, including the current study, is that clinical surrogate biomarkers are used for increased concentrations of IL-1 during subject selection. Given the clinical and pathophysiological heterogeneity of COPD, it may be more appropriate for future studies to select subjects using direct measurements of the concentrations of the target cytokines. However, analysis of target cytokine concentrations in the blood alone may not accurately represent inflammation occurring in the lungs [44] and can be difficult to detect, while sputum sampling can be variable and challenging in large multicentre studies. Improvements in non-invasive lung sampling would be desirable. These sampling challenges also limited our ability to determine whether IL-1R1 and its downstream pathways were inhibited in the lung by MEDI8968 treatment.

## Conclusions

In conclusion, although MEDI8968 demonstrated an acceptable safety profile, it did not produce statistically significant results for efficacy endpoints and ruled out a reduction in exacerbation rate of more than 32%, despite strong pre-clinical data and efforts to identify a target patient population by segmentation of subjects with COPD into subgroups defined by clinical characteristics or biomarkers. This clinical study raises the possibility that patients with COPD and high neutrophil counts may benefit from inhibition of IL-1R1; however, baseline serum CRP or fibrinogen concentrations may not be suitable biomarkers to identify this population.

## Additional file

Additional file 1: Methods and Results relating to this manuscript. (DOCX 774 kb)

## Abbreviations

AECOPD: Acute exacerbations of chronic obstructive pulmonary disease
AERR: Annual exacerbation rate reduction
CI: Confidence interval
COPD: Chronic obstructive pulmonary disease
CRP: C-reactive protein
E-RS: Exacerbations of Chronic Pulmonary Disease Tool Respiratory Symptoms
FEV_1_: Forced expiratory volume in 1 s
GOLD: Global Initiative for Chronic Obstructive Lung Disease
IL-R1: Interleukin-1 receptor 1
ITT: Intention-to-treat
IV: Intravenous
mITT: Modified intention-to-treat
PK: Pharmacokinetic
Q4W: Every 4 weeks
SC: Subcutaneous
SD: Standard deviation
SGRQ-C: St George’s Respiratory Questionnaire-COPD
TEAE: Treatment-emergent adverse events
TESAE: Treatment-emergent serious adverse event

## References

[1] Global strategy for the diagnosis, management, and prevention of chronic obstructive pulmonary disease. Global Initiative for Chronic Obstructive Lung Disease (GOLD). 2016. http://goldcopd.org/. Accessed 26 Apr 2017.

[2] Global surveillance, prevention and control of chronic respiratory diseases. World Health Organization (WHO). 2007. http://www.who.int/respiratory/publications/global_surveillance/en/. Accessed 26 Apr 2017.

[3] Soler-Cataluña JJ, Martínez-García MÁ, Román Sánchez P, Salcedo E, Navarro M, Ochando R (2005). Severe acute exacerbations and mortality in patients with chronic obstructive pulmonary disease. Thorax.

[4] Caramori G, Adcock IM, Di Stefano A, Chung KF (2014). Cytokine inhibition in the treatment of COPD. Int J Chron Obstruct Pulmon Dis.

[5] Bauer CMT, Morissette MC, Stämpfli MR (2013). The influence of cigarette smoking on viral infections: translating bench science to impact COPD pathogenesis and acute exacerbations of COPD clinically. Chest.

[6] Botelho FM, Bauer CMT, Finch D, Nikota JK, Zavitz CCJ, Kelly A, Lambert KN, Piper S, Foster ML, Goldring JJP (2011). IL-1alpha/IL-1R1 expression in chronic obstructive pulmonary disease and mechanistic relevance to smoke-induced neutrophilia in mice. PLoS One.

[7] Pauwels NS, Bracke KR, Dupont LL, Van Pottelberge GR, Provoost S, Vanden Berghe T, Vandenabeele P, Lambrecht BN, Joos GF, Brusselle GG (2011). Role of IL-1alpha and the Nlrp3/caspase-1/IL-1beta axis in cigarette smoke-induced pulmonary inflammation and COPD. Eur Respir J.

[8] O'Donnell RA, Peebles C, Ward JA, Daraker A, Angco G, Broberg P, Pierrou S, Lund J, Holgate ST, Davies DE (2004). Relationship between peripheral airway dysfunction, airway obstruction, and neutrophilic inflammation in COPD. Thorax.

[9] Sapey E, Bayley D, Ahmad A, Newbold P, Snell N, Stockley RA (2008). Inter-relationships between inflammatory markers in patients with stable COPD with bronchitis: intra-patient and inter-patient variability. Thorax.

[10] Hacievliyagil SS, Gunen H, Mutlu LC, Karabulut AB, Temel I (2006). Association between cytokines in induced sputum and severity of chronic obstructive pulmonary disease. Respir Med.

[11] Bafadhel M, McKenna S, Terry S, Mistry V, Reid C, Haldar P, McCormick M, Haldar K, Kebadze T, Duvoix A (2011). Acute exacerbations of chronic obstructive pulmonary disease: identification of biologic clusters and their biomarkers. Am J Respir Crit Care Med.

[12] Castro P, Legora-Machado A, Cardilo-Reis L, Valenca S, Porto LC, Walker C, Zuany-Amorim C, Koatz VLG (2004). Inhibition of interleukin-1beta reduces mouse lung inflammation induced by exposure to cigarette smoke. Eur J Pharmacol.

[13] Sethi S, Maloney J, Grove L, Wrona C, Berenson CS (2006). Airway inflammation and bronchial bacterial colonization in chronic obstructive pulmonary disease. Am J Respir Crit Care Med.

[14] Sethi S, Murphy TF (2008). Infection in the pathogenesis and course of chronic obstructive pulmonary disease. N Engl J Med.

[15] Hernandez ML, Mills K, Almond M, Todoric K, Aleman MM, Zhang H, Zhou H, Peden DB (2015). IL-1 receptor antagonist reduces endotoxin-induced airway inflammation in healthy volunteers. J Allergy Clin Immunol.

[16] Cardiel MH, Tak PP, Bensen W, Burch FX, Forejtova S, Badurski JE, Kakkar T, Bevirt T, Ni L, McCroskery E (2010). A phase 2 randomized, double-blind study of AMG 108, a fully human monoclonal antibody to IL-1R, in patients with rheumatoid arthritis. Arthritis Res Ther..

[17] Cohen SB, Proudman S, Kivitz AJ, Burch FX, Donohue JP, Burstein D, Sun YN, Banfield C, Vincent MS, Ni L, Zack DJ (2011). A randomized, double-blind study of AMG 108 (a fully human monoclonal antibody to IL-1R1) in patients with osteoarthritis of the knee. Arthritis Res Ther.

[18] Anthonisen NR, Manfreda J, Warren CPW, Hershfield ES, Harding GK, Nelson NA (1987). Antibiotic therapy in exacerbations of chronic obstructive pulmonary disease. Ann Intern Med.

[19] Safety and efficacy of multiple doses of canakinumab (ACZ885) in chronic obstructive pulmonary disease (COPD) patients. Novartis. 2011. http://clinicaltrials.gov/show/NCT00581945. Accessed 26 Apr 2017.

[20] Jones PW, Quirk FH, Baveystock CM (1991). The St George's respiratory questionnaire. Respir Med.

[21] Meguro M, Barley EA, Spencer S, Jones PW (2007). Development and validation of an improved, COPD-specific version of the St. George Respir Questionnaire Chest.

[22] Boxer LA (2012). How to approach neutropenia. Hematology Am Soc Hematol Educ Program..

[23] Furst DE (2010). The risk of infections with biologic therapies for rheumatoid arthritis. Semin Arthritis Rheum.

[24] Salliot C, Dougados M, Gossec L (2009). Risk of serious infections during rituximab, abatacept and anakinra treatments for rheumatoid arthritis: meta-analyses of randomised placebo-controlled trials. Ann Rheum Dis.

[25] Rennard SI, Calverley PMA, Goehring UM, Bredenbröker D, Martinez FJ (2011). Reduction of exacerbations by the PDE4 inhibitor roflumilast--the importance of defining different subsets of patients with COPD. Respir Res.

[26] Anzueto A, Miravitlles M (2009). Efficacy of tiotropium in the prevention of exacerbations of COPD. Ther Adv Respir Dis.

[27] Miller BE, Tal-Singer R, Rennard SI, Furtwaengler A, Leidy N, Lowings M, Martin UJ, Martin TR, Merrill DD, Snyder J (2016). Plasma fibrinogen qualification as a drug development tool in chronic obstructive pulmonary disease. Perspective of the chronic obstructive pulmonary disease biomarker qualification consortium. Am J Respir Crit Care Med.

[28] Deng ZC, Zhao P, Cao C, Sun SF, Zhao F, Lu CY, Ma HY (2014). C-reactive protein as a prognostic marker in chronic obstructive pulmonary disease. Exp Ther Med.

[29] Verweij CL, Higgs BW, Yao Y. Personalized healthcare in autoimmune diseases. In: Yao Y, Jallal B, Ranade K, editors. Genomic biomarkers for pharmaceutical development. Elsevier; 2014. p. 51–71.

[30] Hurst JR, Vestbo J, Anzueto A, Locantore N, Müllerova H, Tal-Singer R, Miller B, Lomas DA, Agusti A, Macnee W (2010). Susceptibility to exacerbation in chronic obstructive pulmonary disease. N Engl J Med.

[31] Thomsen M, Ingebrigtsen TS, Marott JL, Dahl M, Lange P, Vestbo J, Nordestgaard BG (2013). Inflammatory biomarkers and exacerbations in chronic obstructive pulmonary disease. JAMA.

[32] Huang YJ, Sethi S, Murphy T, Nariya S, Boushey HA, Lynch SV (2014). Airway microbiome dynamics in exacerbations of chronic obstructive pulmonary disease. J Clin Microbiol.

[33] Singh R, Mackay AJ, Patel ARC, Garcha DS, Kowlessar BS, Brill SE, Donnelly LE, Barnes PJ, Donaldson GC, Wedzicha JA (2014). Inflammatory thresholds and the species-specific effects of colonising bacteria in stable chronic obstructive pulmonary disease. Respir Res.

[34] Peng C, Tian C, Zhang Y, Yang X, Feng Y, Fan H (2013). C-reactive protein levels predict bacterial exacerbation in patients with chronic obstructive pulmonary disease. Am J Med Sci.

[35] Sethi S, Wrona C, Eschberger K, Lobbins P, Cai X, Murphy TF (2008). Inflammatory profile of new bacterial strain exacerbations of chronic obstructive pulmonary disease. Am J Respir Crit Care Med.

[36] Chapman RW, Minnicozzi M, Celly CS, Phillips JE, Kung TT, Hipkin RW, Fan X, Rindgen D, Deno G, Bond R (2007). A novel, orally active CXCR1/2 receptor antagonist, Sch527123, inhibits neutrophil recruitment, mucus production, and goblet cell hyperplasia in animal models of pulmonary inflammation. J Pharmacol Exp Ther.

[37] Chapman RW, Phillips JE, Hipkin RW, Curran AK, Lundell D, Fine JS (2009). CXCR2 antagonists for the treatment of pulmonary disease. Pharmacol Ther.

[38] Dhimolea E (2010). Canakinumab. MAbs.

[39] Kaur M, Singh D (2013). Neutrophil chemotaxis caused by chronic obstructive pulmonary disease alveolar macrophages: the role of CXCL8 and the receptors CXCR1/CXCR2. J Pharmacol Exp Ther.

[40] Mahler DA, Huang S, Tabrizi M, Bell GM (2004). Efficacy and safety of a monoclonal antibody recognizing interleukin-8 in COPD: a pilot study. Chest.

[41] Matera MG, Calzetta L, Cazzola M (2010). TNF-alpha inhibitors in asthma and COPD: we must not throw the baby out with the bath water. Pulm Pharmacol Ther.

[42] Rennard SI, Fogarty C, Kelsen S, Long W, Ramsdell J, Allison J, Mahler D, Saadeh C, Siler T, Snell P (2007). The safety and efficacy of infliximab in moderate to severe chronic obstructive pulmonary disease. Am J Respir Crit Care Med.

[43] Gernez Y, Tirouvanziam R, Chanez P (2010). Neutrophils in chronic inflammatory airway diseases: can we target them and how?. Eur Respir J.

[44] Röpcke S, Holz O, Lauer G, Müller M, Rittinghausen S, Ernst P, Lahu G, Elmlinger M, Krug N, Hohlfeld JM (2012). Repeatability of and relationship between potential COPD biomarkers in bronchoalveolar lavage, bronchial biopsies, serum, and induced sputum. PLoS One.

