# Supplementary material for: A randomised, placebo-controlled trial of anti–interleukin-1 receptor 1 monoclonal antibody MEDI8968 in chronic obstructive pulmonary disease
Source: Respir Res. 2017 Aug 9;18:153. doi: 10.1186/s12931-017-0633-7 (PMC5551010; doi:10.1186/s12931-017-0633-7)
Supplement: Additional file 1: — Methods and Results relating to this manuscript. (DOCX 774 kb) [file 12931_2017_633_MOESM1_ESM.docx]

Supplemental information

A randomised, placebo-controlled trial of anti–interleukin-1 receptor 1 monoclonal antibody MEDI8968 in chronic obstructive pulmonary disease

Peter M. A. Calverley, Sanjay Sethi, Michelle Dawson, Christine K. Ward, Donna K. Finch, Mark Penney, Paul Newbold, and René van der Merwe

# Table of contents

[Methods 4](#_Toc480464863)

[Full inclusion criteria 4](#_Toc480464864)

[Full exclusion criteria 5](#_Toc480464865)

[Study-stopping criteria 8](#_Toc480464866)

[Maintenance therapy by baseline FEV_1_ 9](#_Toc480464867)

[Randomisation and blinding 9](#_Toc480464868)

[Pharmacokinetic and immunogenic profile measurements 10](#_Toc480464869)

[Exploratory endpoints 10](#_Toc480464870)

[Safety and tolerability 11](#_Toc480464871)

[Results 12](#_Toc480464872)

[Immunogenicity profile 12](#_Toc480464873)

[Mean (SD) SGRQ-C scores 12](#_Toc480464874)

[Change from baseline in SGRQ-C scores 13](#_Toc480464875)

[Change from baseline in E-RS total score 13](#_Toc480464876)

[Change from baseline in SGRQ-C total score by neutrophil tertile 13](#_Toc480464877)

[Moderate/severe AECOPD rate by subgroup 13](#_Toc480464878)

[Change from baseline in FEV_1_ 13](#_Toc480464879)

[Summary of TEAEs 13](#_Toc480464880)

[Figure E1 14](#_Toc480464881)

[Figure E2 15](#_Toc480464882)

[Figure E3 16](#_Toc480464883)

[Table E1 17](#_Toc480464884)

[Table E2 18](#_Toc480464885)

[Table E3 19](#_Toc480464886)

# Methods

## Full inclusion criteria

Full inclusion criteria included:

- Age 45–75 years inclusive at the time of screening
- Written informed consent and any locally required authorisation (e.g. Health Insurance Portability and Accountability Act [HIPAA] in the USA, EU Data Privacy Directive in the EU) obtained from the subject prior to performing any protocol-related procedures, including screening evaluations
- Body mass index between 16 and 35 kg/m^2^ at screening
- Able to read and write and use the electronic devices required for the study
- Chronic obstructive pulmonary disease (COPD) with a post-bronchodilator forced expiratory volume in 1 second (FEV_1_)/forced vital capacity <0.70 and a post‑bronchodilator FEV_1_ <80% predicted (Global Initiative for Chronic Obstructive Lung Disease [GOLD] stage II, III and IV) at screening
- Evidence of COPD symptoms defined as the daily sum of three items in the EXAcerbations of Chronic pulmonary disease Tool (EXACT; breathlessness, cough, and sputum quantity) score of ≥2 for 7 of the last 14 days prior to randomisation (day 1)
- Documented history of two or more acute exacerbations of COPD (AECOPD) 12 months prior to screening or three or more AECOPD 18 months prior to screening that required treatment with oral corticosteroids, and/or antibiotics, and/or required an emergency room visit and/or hospitalisation
- Clinically stable and free from an AECOPD for 8 weeks prior to day 1
- Current smoker or ex-smoker with a tobacco history of ≥10 pack-years (1 pack‑year = 20 cigarettes smoked per day for 1 year)
- Females of childbearing potential who were sexually active with a non-sterilised male partner had to use highly effective contraception from screening, and had to agree to continue using such precautions through the end of the study
- Non-sterilised males or sterilised males who had <1 year post-vasectomy and who were sexually active with a female partner of childbearing potential had to use a highly effective method of contraception from day 1 through the end of the study

## Full exclusion criteria

Full exclusion criteria included:

- Past or present disease or disorder which, as judged by the investigator and the medical monitor, might have either put the subject at risk because of participation in the study, or might have affected the outcome of this study
- Concurrent enrolment in another clinical study
- Employees of the clinical study site or any other individuals involved with the conduct of the study, or immediate family members of such individuals
- Significant or unstable ischaemic heart disease, arrhythmia, cardiomyopathy, heart failure, renal failure, uncontrolled hypertension as defined by the investigator, or any other relevant cardiovascular disorder as judged by the investigator that might have either put the subject at risk because of participation in the study, or might have affected the outcome of this study
- Pregnant or breastfeeding women
- Known history of allergy or reaction to any component of the investigational product formulation or to any other biologic therapy, or budesonide/formoterol preparations, tiotropium bromide, or salbutamol (albuterol) or any of their excipients
- Other significant pulmonary disease as a primary diagnosis (e.g. cystic fibrosis, bronchiectasis, alpha-1 antitrypsin deficiency, interstitial lung disease; pulmonary hypertension other than cor pulmonale). If a subject is diagnosed with any other pulmonary disease as a secondary diagnosis, he/she might have been included if, in the opinion of the investigator or medical monitor, the inclusion of the subject did not compromise the interpretation of the study
- Receiving long-term oxygen therapy (LTOT) at screening; LTOT defined as >15 hours/day
- Subjects who had a tracheostomy *in situ*
- Subjects with lung volume reduction surgery within the 12 months prior to screening
- Past or current malignancy within the past 5 years, except for adequately treated non-invasive basal cell and squamous cell carcinoma of the skin and cervical carcinoma *in situ* treated with apparent success more than 1 year prior to screening
- Subjects who had a chest X-ray (posterior-anterior and lateral) or computed tomography (CT) scan within the previous 12 months prior to screening with findings suggestive of malignancy or tuberculosis (TB). The investigator had to review both actual chest X-ray/CT scan and the associated report. If a historical chest X-ray or CT scan had not been performed within the previous 12 months, an assessment was conducted at screening
- Subjects who, in the opinion of the investigator or qualified designee, had evidence of active TB, either treated or untreated, or latent TB without completion of an appropriate course of treatment or appropriate ongoing prophylactic treatment
- Fever >38.0 °C (>100.4 °F) at screening and/or day 1 measured using oral temperature (or equivalent)
- Presence of a serious infection, defined as requiring hospitalisation or recurrent, acute or chronic infections within 8 weeks prior to screening. Subjects with a history of osteomyelitis were excluded
- Use of immunosuppressive medication, including oral and/or other systemic corticosteroids within 8 weeks prior to randomisation (day 1) into the study
- Receipt of any biologic agent or novel investigational medicinal product within 3 months or 5 half-lives prior to screening, whichever was greater, and through to study end
- Participation in, or scheduled for, an intensive COPD rehabilitation programme (subjects who were in the maintenance phase of a rehabilitation programme were eligible to take part)
- Current diagnosis of asthma according to Global Initiative for Asthma (GINA) guidelines
- Previous treatment with MEDI8968/AMG108
- An absolute neutrophil count of <2.5 × 10^3^ cells/μL
- Subjects who had received a live or live attenuated vaccine within 4 weeks prior to screening
- Positive hepatitis B surface antigen or hepatitis C virus antibody serology. Subjects with a history of hepatitis B vaccination without a history of hepatitis B were allowed to participate
- A positive human immunodeficiency virus test at screening or subject having taken anti-retroviral medications, as determined by medical history and/or subject’s verbal report
- Major surgery within 3 weeks prior to screening, or planned in-patient surgery or hospitalisation during the study period
- History of chronic alcohol or drug abuse within 12 months prior to screening, or any condition associated with poor compliance as judged by the investigator
- Uncontrolled, clinically significant history of liver disease or elevated aspartate transaminase or alanine transaminase >1.5 × upper limit of normal at screening

## Study-stopping criteria

Study-stopping criteria included:

- Death of any subject in which the cause of death is assessed as related to the investigational product
- Anaphylactic reaction (immediate life-threatening allergic reaction with bronchoconstriction, angioedema and/or hypotension) requiring epinephrine
- Infusion reaction that includes signs and symptoms indicative of an anaphylactoid reaction. These usually develop within 2 hours after the start of investigational product administration and often resolve within 24 hours after completion of investigational product administration. Signs/symptoms may include the following: urticaria, arthralgia, bronchospasm, wheeze, cough, dizziness, dyspnoea, fatigue, headache, hypertension, hypotension, myalgia and vomiting
- Immune complex disease with manifestations such as arthralgias, serum-sickness and vasculitis autoimmunity

## Maintenance therapy by baseline FEV_1_

Subjects with FEV_1_ <50% predicted received budesonide/formoterol 160/4.5 μg (two inhalations a day); among these subjects, those receiving tiotropium prior to study entry continued this concomitant therapy (18 μg/inhalation, once daily). Subjects with FEV_1_ ≥50–<80% predicted received tiotropium 18 μg/inhalation once daily; if these subjects were previously on budesonide/formoterol prior to screening, this was discontinued.

## Randomisation and blinding

Subjects who were enrolled by the investigator were randomised in a 1:1 ratio via an interactive voice/web response system. Randomisation was restricted to ensure that approximately 80% of subjects were GOLD stage III–IV (severe-to-very severe disease) and was stratified by background maintenance therapy designated during screening (tiotropium, budesonide/formoterol 160/4.5 μg or tiotropium with budesonide/formoterol 160/4.5 μg). The restricted randomisation ensured a study population that was representative of the target population for this therapy.

MEDI8968 and placebo are distinguishable in appearance. Investigational product was handled by an unblinded investigational product manager at the site and was administered by an unblinded study team member who was not involved in the management of study subjects. Neither the subjects nor the investigators or sponsor staff who were involved in the treatment or clinical evaluation of the subjects were aware of the treatment. An independent investigational product monitor was also unblinded to perform investigational product accountability.

## Pharmacokinetic and immunogenic profile measurements

The pharmacokinetic (PK) population included the subjects who were randomised and received at least one dose of investigational product and had at least one post‑dose serum concentration measurement above the lower limit of quantitation. Blood samples for MEDI8968 concentration determination were collected at weeks 1, 4, 5, 13, 25, 37, 53, 61 and 69. Serum MEDI8968 exposure was measured using a validated enzyme-linked immunosorbent assay (ELISA) utilising interleukin‑1 receptor 1 (IL‑1R1) as the capture receptor and biotinylated IL-1R1 for detection; the lower limit of quantitation was 78.13 ng/mL.

The immunogenicity population included the subjects who were randomised and received at least one dose of investigational product and had at least one post-dose serum sample for immunogenicity testing. The presence of anti-drug antibodies (ADAs) was evaluated in serum at weeks 1, 5, 13, 25, 37, 53, 61 and 69. ADAs were detected using an electrochemiluminescence (ECL) based bridging immunoassay to screen for and confirm antibodies capable of binding MEDI8968. PK and immunogenic (IM) samples collected on a dosing visit were sampled pre‑dose.

## Exploratory endpoints

Change from baseline in pre-bronchodilator FEV_1_ was measured at weeks 1, 13, 25, 37, 53, 61 and 69, and change from baseline in the Exacerbations of Chronic Pulmonary Disease Tool-Respiratory Symptoms (E-RS) total score was evaluated based on the EXACT daily diary.

## Safety and tolerability

Safety and tolerability were assessed by recording treatment-emergent adverse events, treatment-emergent serious adverse events, laboratory measurements, vital signs and electrocardiograms. Neutrophil counts were also monitored at each clinic visit in all study subjects and subjects followed a schedule of dosing restrictions and follow-up requirements if they fell below 1.5 × 10^3^ cells/μL.

# Results

## Immunogenicity profile

The immunogenicity population included a total of 320 subjects (*n* = 163 placebo; *n* = 157 MEDI8968). Of the 155 MEDI8968-dosed subjects from whom ADA samples had been collected, 19 (12.3%) were confirmed positive for ADAs (post-baseline). Of those confirmed positive, nine subjects were judged to have neutralising ADAs due to a temporal correlation between the presence of ADAs and MEDI8968 serum concentrations below the limit of quantitation. Ten (6.2%) placebo-treated subjects tested positive for ADAs, consistent with the 5% false-positive cutpoint of the ADA assay. There was no apparent relationship between ADA titres and effect on PK exposure or any treatment effect. Although analysis of the data excluding subjects with neutralising ADAs was not performed, the exacerbation rate in these subjects was slightly lower compared with other subjects in the MEDI8968 group. If these subjects were excluded from the analysis, the exacerbation rate in the active arm would therefore increase slightly and the conclusions would remain the same.

## Mean (SD) SGRQ-C scores

Mean (standard deviation [SD]) St George’s Respiratory Questionnaire-chronic obstructive pulmonary disease (SGRQ-C) total score at week 53 was 55.24 (17.92) with placebo and 56.91 (18.93) with MEDI8968. The proportion of subjects experiencing a 4-, 8- or 12-point improvement in SGRQ-C total score was similar between treatment groups at all timepoints analysed. Mean (SD) SGRQ-C symptom domain score at week 53 was 65.74 (18.18) with placebo and 65.60 (19.63) with MEDI8968.

## Change from baseline in SGRQ-C scores

From week 13 until the end of the study, MEDI8968 resulted in smaller, non-statistically significant reductions in SGRQ-C total score from baseline than placebo (Fig. E1A). In contrast to the results for the total score, the MEDI8968 group experienced numerically larger reductions from baseline in the SGRQ-C symptom domain score than the placebo group from week 5 to week 53 (Fig. E1B); however, these results were not statistically significant.

## Change from baseline in E-RS total score

The MEDI8968 group experienced numerically greater reductions from baseline in the E-RS total score than the placebo group (Fig. E2); however, these results were not statistically significant.

## Change from baseline in SGRQ-C total score by neutrophil tertile

There were no statistically significant changes from baseline in SGRQ-C total score by neutrophil tertile between treatment groups at any timepoint analysed (Fig. E3).

## Moderate/severe AECOPD rate by subgroup

Table E1 shows the moderate/severe AECOPD rate by subgroup analysis.

## Change from baseline in FEV_1_

There were no statistically significant changes from baseline in FEV_1_ between treatment groups at any time point analysed (Table E2).

## Summary of TEAEs

Table E3 shows an overview of the treatment-emergent adverse events (TEAEs) and the most common TEAEs experienced in ≥3% of subjects in the MEDI8968 group.

## Fig. E1 SGRQ-C change from baseline over time in (A) total score and (B) symptom domain score (mITT population).*


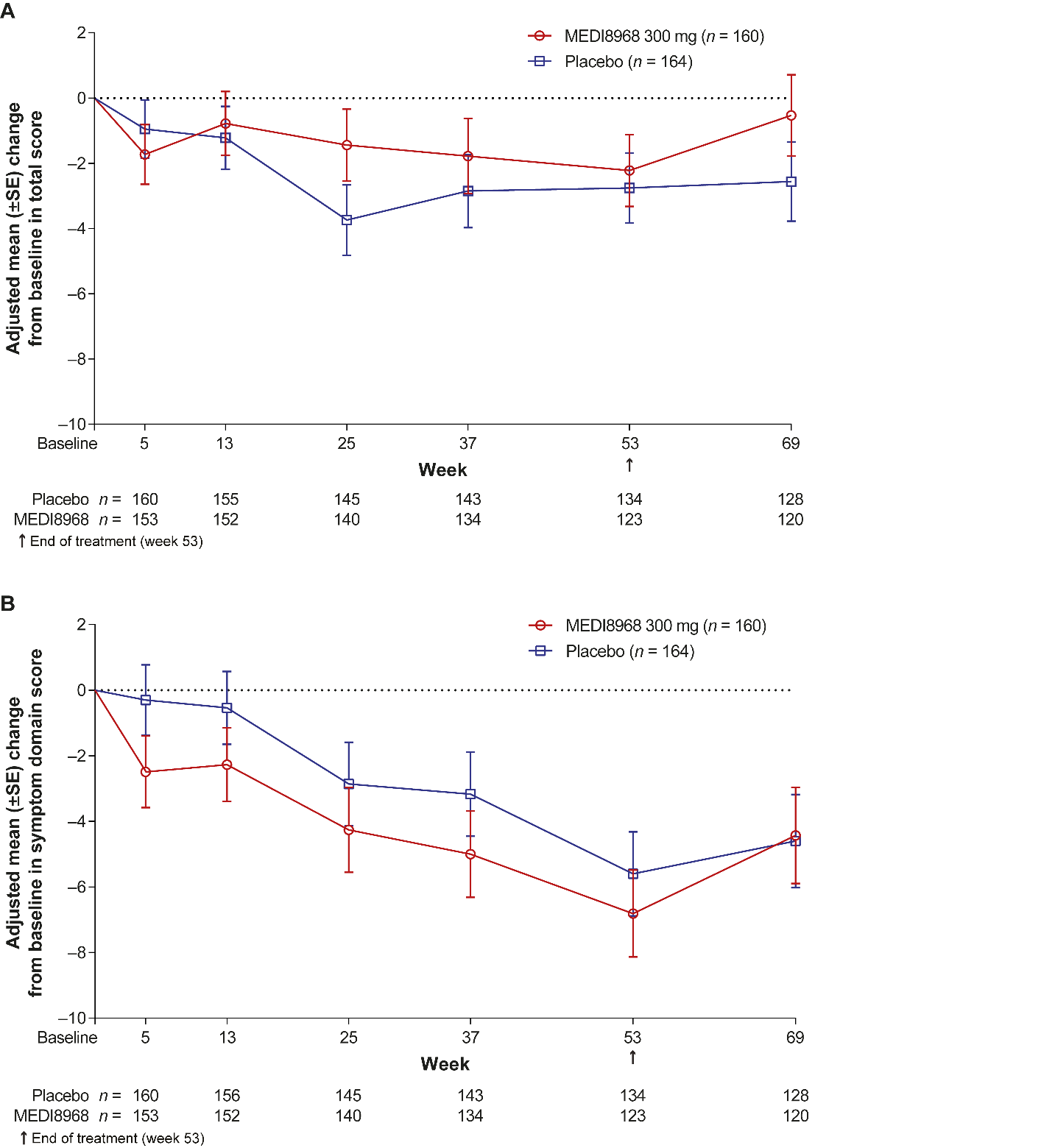


*Repeated measures analysis of change from baseline = baseline + treatment + visit + background therapy + treatment*visit. Correlation between visits within a subject modelled using an unstructured covariance matrix.

mITT: modified intention-to-treat. SE: standard error. SGRQ-C: St George’s Respiratory Questionnaire-chronic obstructive pulmonary disease.

## Fig. E2 E-RS total score change from baseline over time (mITT population).*


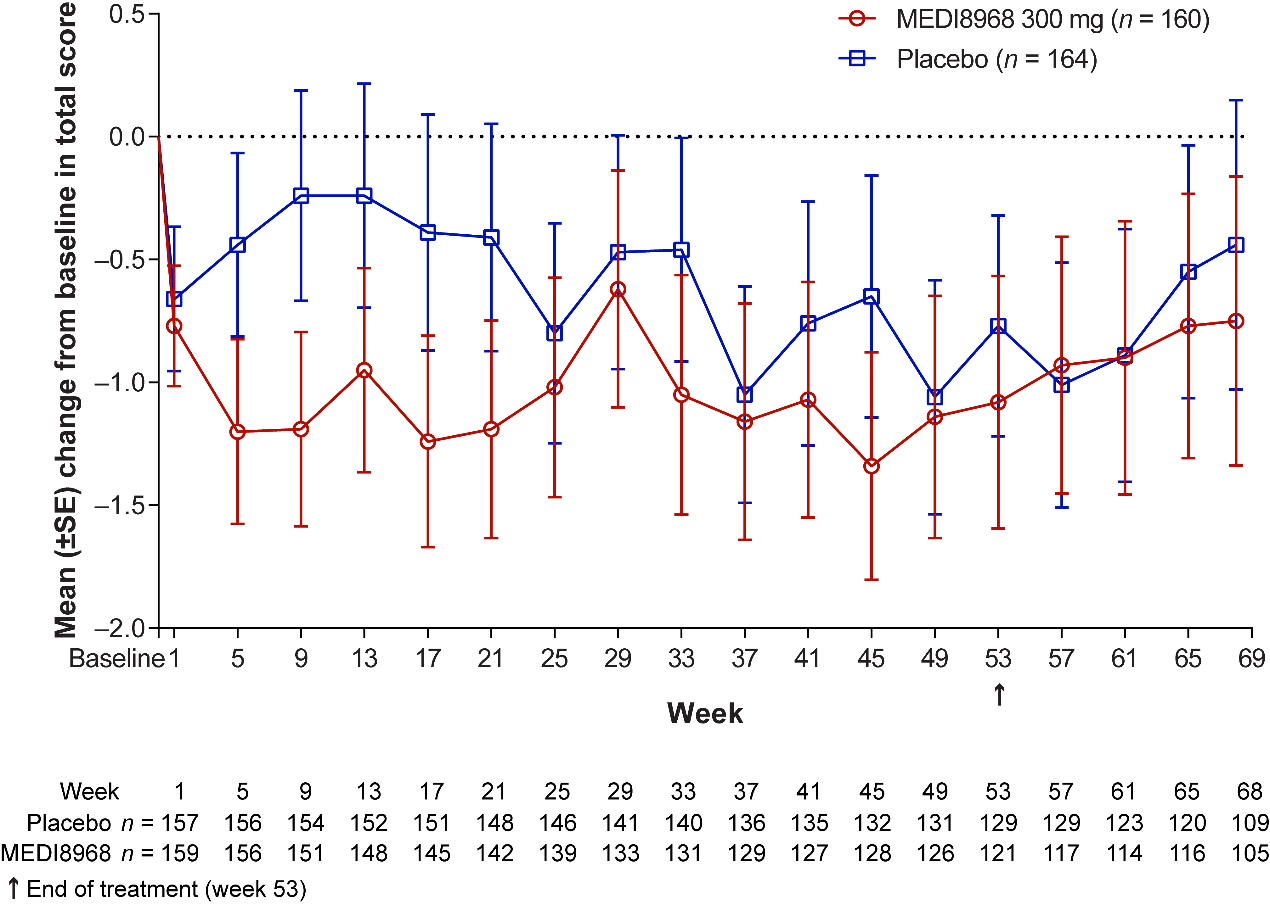


*Raw mean and standard errors estimated at each visit. Change from baseline = visit value – baseline value.

E-RS: Exacerbations of chronic pulmonary disease tool-Respiratory Symptoms. mITT: modified intention-to-treat. SE: standard error.

## Fig. E3 Adjusted mean change from baseline in SGRQ-C total score by neutrophil tertile: (A) 1st tertile; (B) 2nd tertile; (C) 3rd tertile.*


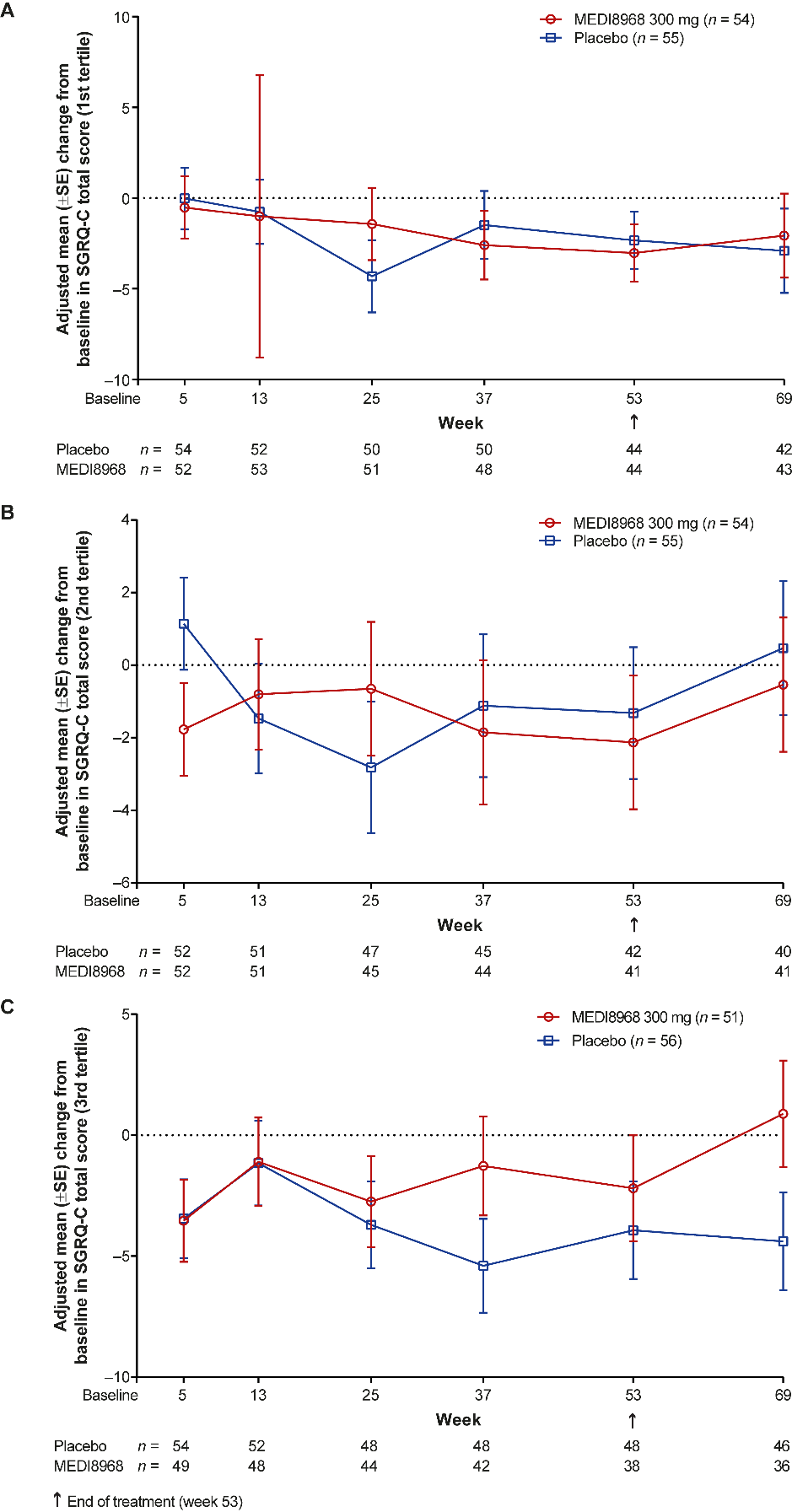


*Repeated measures analysis of change from baseline = baseline + treatment + visit + background therapy + treatment*visit. Correlation between visits within a subject modelled using an unstructured covariance matrix.

SE: standard error. SGRQ-C: St George’s Respiratory Questionnaire-chronic obstructive pulmonary disease.

## Table E1 Moderate/severe AECOPD rate by subgroup (mITT population).

| **Subgroup** | **Treatment group** | **Mean (90% CI) AECOPD rate*** | **Treatment ratio (90% CI)*** | ***P*-value*** |
| --- | --- | --- | --- | --- |
| CRP <0.347 mg/dL | Placebo  (*n* = 84)  MEDI8968  (*n* = 84) | 0.92  (0.68, 1.23)  0.61  (0.43, 0.87) | 0.67 (0.43, 1.04) | 0.135 |
| CRP ≥0.347 mg/dL | Placebo  (*n* = 80)  MEDI8968  (*n* = 76) | 0.60  (0.44, 0.82)  0.80  (0.60, 1.06) | 1.34 (0.88, 2.04) | 0.257 |
| Fibrinogen < median | Placebo  (*n* = 82)  MEDI8968  (*n* = 73) | 0.70  (0.49, 1.02)  0.51  (0.33, 0.78) | 0.72 (0.41, 1.25) | 0.323 |
| Fibrinogen ≥ median | Placebo  (*n* = 77)  MEDI8968  (*n* = 85) | 0.79  (0.60, 1.05)  0.90  (0.70, 1.17) | 1.14 (0.79, 1.65) | 0.558 |
| 1st neutrophil tertile^†^ (≤4.27 × 10^3^ cells/μL) | Placebo  (*n* = 55)  MEDI8968  (*n* = 54) | 0.73  (0.47, 1.11)  0.69  (0.45, 1.08) | 0.96 (0.53, 1.72) | 0.903 |
| 2nd neutrophil tertile^†^ (>4.27 × 10^3^–≤5.68 × 10^3^ cells/μL) | Placebo  (*n* = 53)  MEDI8968  (*n* = 55) | 0.58  (0.39, 0.84)  0.53  (0.35, 0.78) | 0.91 (0.54, 1.54) | 0.775 |
| 3rd neutrophil tertile^†^ (>5.68 × 10^3^ cells/μL) | Placebo  (*n* = 56)  MEDI8968  (*n* = 51) | 0.93  (0.69, 1.25)  0.77  (0.55, 1.08) | 0.83 (0.55, 1.26) | 0.456 |

*Analysed using Poisson regression with Pearson correction, adjusting for treatment, background therapy and history of previous exacerbations.

^†^*Post-hoc* analysis.

AECOPD: acute exacerbations of chronic obstructive pulmonary disease. CI: confidence interval. CRP: C-reactive protein. mITT: modified intention-to-treat.

## Table E2 Adjusted mean change from baseline in pre-bronchodilator FEV_1_ (mITT population).

|  | **Treatment group** | **Mean change from baseline at week 53 (SE)** | **MEDI8968 *vs.* placebo (SE)** | **90% CI** | ***P-*value*** |
| --- | --- | --- | --- | --- | --- |
| Week 13 | Placebo | –0.003 (0.017) | 0.005 (0.024) | (–0.035, 0.045) | 0.833 |
|  | MEDI8968 | 0.002 (0.017) |  |  |  |
| Week 25 | Placebo | –0.018 (0.017) | 0.000 (0.025) | (–0.041, 0.041) | 0.999 |
|  | MEDI8968 | –0.018 (0.018) |  |  |  |
| Week 37 | Placebo | –0.030 (0.018) | 0.031 (0.026) | (–0.012, 0.073) | 0.232 |
|  | MEDI8968 | 0.001 (0.018) |  |  |  |
| Week 53 | Placebo | –0.027 (0.023) | 0.006 (0.033) | (–0.048, 0.060) | 0.851 |
|  | MEDI8968 | –0.021 (0.024) |  |  |  |
| Week 61 | Placebo | –0.029 (0.020) | –0.005 (0.029) | (–0.053, 0.044) | 0.870 |
|  | MEDI8968 | –0.034 (0.021) |  |  |  |
| Week 69 | Placebo | –0.049 (0.021) | 0.018 (0.031) | (–0.033, 0.069) | 0.561 |
|  | MEDI8968 | –0.032 (0.022) |  |  |  |

*Analysed using repeated measures.

CI: confidence interval. FEV_1_: forced expiratory volume in 1 second. mITT: modified intention-to-treat. SE: standard error.

## Table E3 Summary of TEAEs (safety population).

|  | **Placebo**  **(*n* = 164)** | **MEDI8968**  **(*n* = 160)** |
| --- | --- | --- |
| Total number of TEAEs | 592 | 567 |
| Subjects reporting ≥1 TEAE, *n* (%) | 130 (79.3) | 130 (81.3) |
| Number of TESAEs | 64 | 66 |
| Subjects reporting ≥1 TESAE, *n* (%) | 35 (21.3) | 41 (25.6) |
| Subjects with TEAEs leading to discontinuation, *n* (%) | 11 (6.7) | 9 (5.6) |
| **TEAEs (preferred term) for ≥3% of subjects in the MEDI8968 group*, *n* (%)** | | |
| Cardiac disorders |  |  |
| Atrial fibrillation | 4 (2.4) | 5 (3.1) |
| Gastrointestinal disorders | | |
| Abdominal pain, upper | 1 (0.6) | 5 (3.1) |
| Nausea | 3 (1.8) | 5 (3.1) |
| General disorders and administration-site conditions | | |
| Pyrexia | 6 (3.7) | 8 (5.0) |
| Infections | | |
| Bronchitis | 4 (2.4) | 5 (3.1) |
| Nasopharyngitis | 11 (6.7) | 11 (6.9) |
| Pharyngitis | 1 (0.6) | 5 (3.1) |
| Pneumonia | 8 (4.9) | 10 (6.3) |
| Upper respiratory tract infection | 7 (4.3) | 5 (3.1) |
| Urinary tract infection | 5 (3.0) | 5 (3.1) |
| Musculoskeletal and connective tissue disorders | | |
| Arthralgia | 2 (1.2) | 5 (3.1) |
| Nervous system disorders | | |
| Headache | 7 (4.3) | 10 (6.3) |
| Respiratory, thoracic and mediastinal disorders | | |
| COPD | 76 (46.3) | 70 (43.8) |
| Dyspnoea | 6 (3.7) | 5 (3.1) |
| Vascular disorders | | |
| Hypertension | 7 (4.3) | 5 (3.1) |

*Subjects were counted only once for each preferred term, regardless of how many events the subject had experienced.

COPD: chronic obstructive pulmonary disease. TEAE: treatment-emergent adverse event. TESAE: treatment-emergent serious adverse event.
